# Wnt16 signaling in bone homeostasis and osteoarthristis

**DOI:** 10.3389/fendo.2022.1095711

**Published:** 2022-12-22

**Authors:** Xiaoping Ye, Xianwen Liu

**Affiliations:** Department of Oral and Maxillofacial Surgery, Stomatological Hospital, Southern Medical University, Guangzhou, China

**Keywords:** Wnt16, bone homeostasis, osteoblast, osteoclast, osteoarthritis, chondrocytes

## Abstract

Wnts are secreted cysteine-rich glycoproteins involved in joint development and skeletal homeostasis and have been implicated in the occurrence of osteoarthritis. Over the past decade, Wnt16, a member of the Wnt family, has received widespread attention for its strong association with bone mineral density, cortical bone thickness, bone strength, and osteoporotic fracture risk. In recent years, further studies have shed light on the role of Wnt16 a positive regulator of bone mass and protective regulator of osteoarthritis progression. Transduction mechanisms and crosstalk involving Wnt16 signaling have also been illustrated. More importantly, local Wnt16 treatment has been shown to ease osteoarthritis, inhibit bone resorption, and promote new bone formation in bone defect models. Thus, Wnt16 is now a potential therapeutic target for skeletal diseases and osteoarthritis. This paper reviews our current understanding of the mechanisms by which Wnt16 signaling regulates bone homeostasis and osteoarthritis.

## Introduction

Bone is originally formed through the process of bone modeling which is a lifelong process beginning during development. There are two modes of bone modeling: intramembranous ossification, which applies to the craniofacial bone and clavicle, and endochondral ossification, which occurs in limb and trunk bones. Bone remodeling is a dynamic process where mature bone tissue is removed from the skeleton and new bone tissue is formed, mainly regulated by osteoblasts and osteoclasts. During this process, old or damaged bone is resorbed by osteoclasts and replaced with new bone formed by osteoblasts (1–3). Skeletal homeostasis is maintained when a balance between bone resorption and bone formation is reached (4). On the contrary, breakdown of this balance leads to the occurrence of skeletal diseases (5, 6).

Osteoarthritis (OA) is a prevalent chronic joint disease that leads to pain and the loss of joint function. The pathological characteristics of OA are articular cartilage degeneration, subchondral bone hyperplasia, joint edge osteogenesis, synovial tissue inflammation and proliferation, ligament and meniscus degeneration and joint capsule hypertrophy (7, 8). Several pathogenic mechanisms are involved in the mediation of OA, including degradation of extracellular matrix, deficient new matrix formation, cell death, and abnormal hypertrophic differentiation of chondrocytes (9–12).

**Figure 1:**
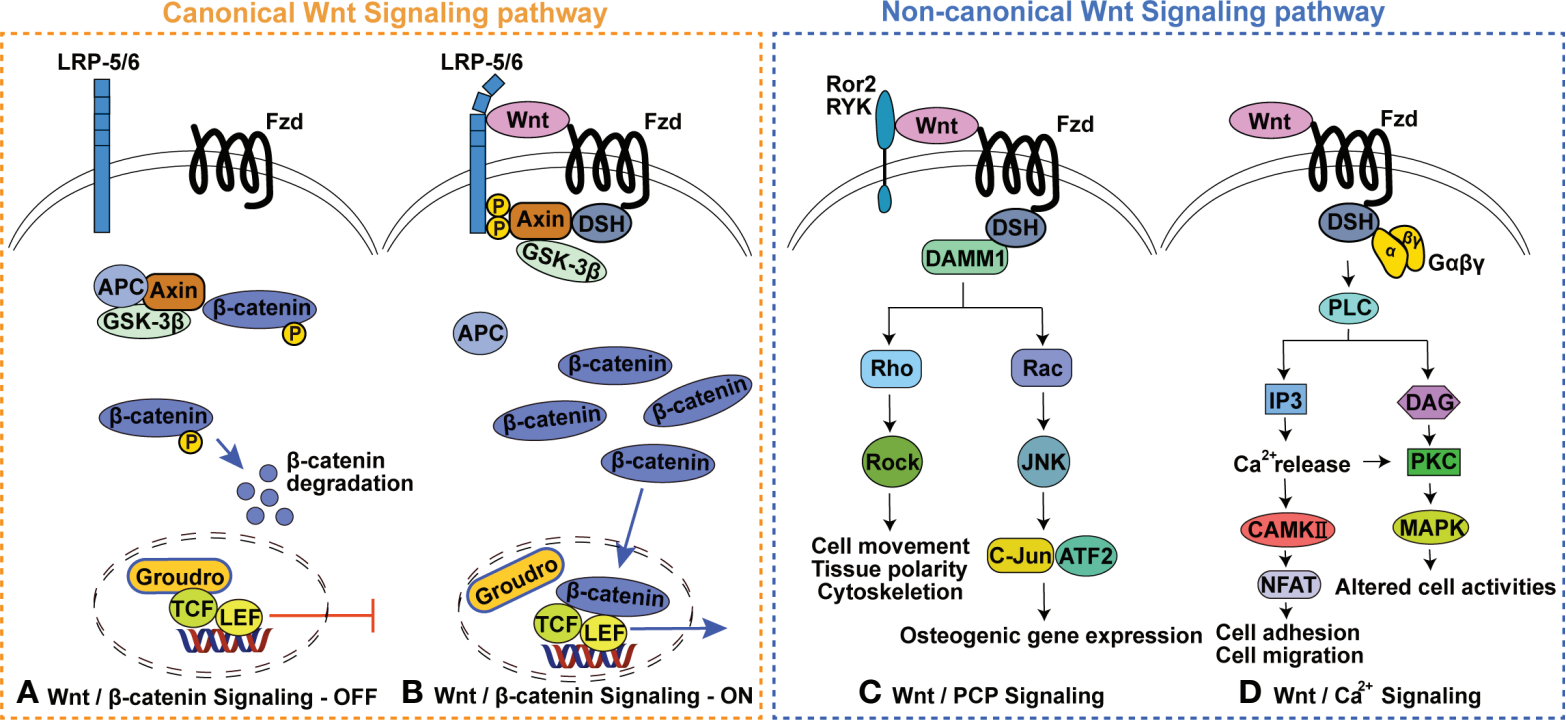
Wnt signaling pathway. **(A)** Wnt/β-catenin Signaling-OFF, **(B)** Wnt/β-catenin Signaling-ON, **(C)** Wnt/planar cell polarity (PCP) Signaling pathway, **(D)** Wnt/Ca^2+^ Signaling pathway. GSK3, glycogen synthase kinase 3; APC, adenomatous polyposis; TCF, T cell factor; LEF, Lymphoid enhancer factor; Fzd, Frizzled receptor protein; LRP5/6, Low-density-lipoprotein-related protein 5/6; DSH, dishevelled; Ror 2, receptor tyrosine Kinase-like Orphan receptors 2; Ryk, Related receptor tyrosine kinase; DAMM1, Disheveled-associated activator of morphogenesis 1; ROCK, Rho activates Rho-associated kinase; JNK, c-Jun N-terminal kinase; PLC, phospholipase C; IP3, Inositol triphosphate; DAG, Diacylglycerol; PKC, Protein kinase C; MAPK, Mitogen-activated protein kinases.

Wnt signaling is subdivided into two branches: canonical Wnt signaling, also known as Wnt/β-catenin signaling, and β-catenin-independent non-canonical Wnt signaling (**Figure 1**) (13–15). The involvement of canonical Wnt signaling in skeletal homeostasis is initially emphasized by the findings that in human loss- and gain-of-function mutations in Low-density-lipoprotein-related protein 5 (LRP5) respectively result in diseases characterized by low bone mass and high bone mass, and loss-of-function mutations in sclerostin cause high bone mass-characterized sclerosteosis and van Buchem disease (16–19). Heterozygous loss-of-function mutations in Wnt1 (the first identified Wnt ligand signaling *via* LRP5/6 in human bone formation) can cause dominantly inherited early-onset osteoporosis, while biallelic loss-of-function mutations lead to recessively inherited osteogenesis imperfecta (20–22). These findings and subsequent studies in genetically modified mouse models have indicated that Wnt signaling is required for all aspects of skeletal development (including bone development, cartilage development and joint formation) (23–27), postnatal bone and joint homeostasis (15, 28–32). Notably, the complex fine nature of the Wnt regulatory network in skeletal homeostasis is highlighted by the findings that both excessive activation and inactivation of Wnt signaling can cause skeletal malformation, bone diseases and cartilage loss (23, 33–39). For example, activation of the classic non-canonical ligand Wnt5a can promote both bone formation and resorption (40–44). Details of this complex network is outside the scope of this review and has been reviewed elsewhere (45–49).

Wnt16 has emerged as a new Wnt ligand strongly associated with bone mineral density (BMD) variations (50–55). Previously, Wnt16 was considered to be a strong determinant of cortical, but not trabecular, bone mass (56). However, subsequent studies have provided new insights on the roles of Wnt16 in both cortical and trabecular bone homeostasis (57–59). Moreover, the role of Wnt16 as a precision regulator in skeletal development and as a protective regulator in OA have also been further illustrated in recent years. Therefore, in this review article, we will focus on reviewing the latest discoveries regarding the role of Wnt16 signaling in skeletal development, bone homeostasis, and OA.

## Regulation of bone and joint development by Wnt16

Current evidence indicates that Wnt16 is involved in both intramembranous and endochondral ossification (60), and its overexpression in chondrocytes attenuates endochondral ossification. Col2a1-Wnt16 transgenic mice, in which Wnt16 is overexpressed in chondrocytes under the control of the Col2a1 promoter and enhancer, exhibit morphological changes involving a significant reduction of tissue mineralization and endochondral ossification during embryonic development (61, 62); this suggests that Wnt16 overexpression during the embryonic period may regulate the positioning and rearrangement of columnar chondrocytes. Further, chondrocyte differentiation and hypertrophy during endochondral ossification are inhibited by Wnt16 overexpression *in vivo* (62).

The congenital deletion of Wnt16 does not appear to have an effect on mouse embryonic development but dose play a role in bone and cartilage homeostasis in later development stage. Systemic and chondrocyte-specific deletion of Wnt16 does not affect endochondral bone formation in mouse embryos, and the morphology, cortical bone parameters, trabecular bone parameters and mechanical parameters of these genetic mice are normal at birth. However, during the process of postnatal skeletal maturation, those mutant mice displayed a smaller body size, lower bone mass, and thinner articular cartilage, suggesting the role of Wnt16 in later development stage (56, 61, 62). Likewise, in zebrafish, congenital Wnt16 deletion can lead to abnormal skeletal development. For instance, knockout of Wnt16 early in zebrafish embryos causes bone deformities of the head, spine, and tail in their adulthood, likely as a result of the down-regulation of genes in the mTOR, FoxO, and VEGF signaling pathways (63). Furthermore, Wnt16 knockout in zebrafish leads to altered jaw joint morphology at 5 days post fertilisation as a result of changes in cell proliferation and migration (64).

These studies demonstrate that appropriate regulation of Wnt16 expression is required for normal bone and joint development, as both excessive and insufficient Wnt16 levels have been shown to cause developmental defects.

## Regulation of bone remodeling by Wnt16

Over the past decade, genome-wide association studies (GWASs) have discovered that missense single-nucleotide polymorphisms (SNPs) in Wnt16 are strongly associated with bone mineral density (BMD) variations at different skeletal sites in different populations, leading to the conclusion that Wnt16 positively affects BMD (50–55). The close association of Wnt16 SNPs with peak bone mass, heel/calcaneal ultrasound parameters, bone geometric parameters (including cortical bone thickness, axial length, neck buckling ratio and cross-sectional area), and fracture risk have also been emphasized (53, 54, 65–68). Notably, despite its success in identifying novel variant-trait associations, GWASs have been largely unsuccessful in assessing the role of low frequency and rare genetic variations. Very recently, two influential GWAS articles filled this gap, highlighting the association of BMD with a novel low-frequency non-coding variant near Wnt16 and rare variants of Wnt16 (69, 70). In addition, as the transcriptional complexity of Wnt16 has been clarified, transcriptome-wide association studies, which are more reliable for interpreting the biological functions of genetic loci than GWASs, have confirmed the association of Wnt16 expression with total body BMD and fracture (71). These new findings provide a more comprehensive picture of the role of Wnt16 in regulating bone homeostasis at the functional level.

Gain- and loss-of-function approaches in mice have contributed to the functional characterization of Wnt16 in cortical bone mass and bone strength. In mice from 5 weeks old to 1 year old, the systemic and osteoblast/chondrocyte-specific deletion of Wnt16 eventually leads to lower bone mass, reduced bone strength (even spontaneous fractures), smaller bone cross-sectional area, and decreased formation of the cortical bone (56, 61, 62, 66, 72); the opposite effects are observed when overexpressing Wnt16 (58, 59). It is clear that Wnt16 plays a role in cortical bone homeostasis during not only development and growth but also aging. Recently, a tamoxifen-inducible Wnt16 inactivation model was used to demonstrate that the effect of Wnt16 on postnatal cortical bone is independent of its effect on skeletal development (73). This new finding undoubtably consolidates the scientific foundation of Wnt16 as a therapeutic target for acquired or age-related bone diseases, such as osteoporosis.

Gain-of-function experiments in mice highlighted the positive regulation of Wnt16 in trabecular bone mass and suggested that a compensatory mechanism to maintain bone strength in the absence of Wnt16 may exist in mice. Studies have shown that overexpressing Wnt16 in osteoblasts or osteocytes increases trabecular bone mass (57–59). Among these studies, two found that the overexpression of Wnt16 in osteoblasts or osteocytes primarily increased trabecular bone mass rather than cortical bone mass, suggesting that trabecular bone may be more sensitive to altered Wnt16 expression (58, 59). In addition, in a mouse calvarial defect model, local transplantation of periosteal derived cells (PDCs), which were genetically engineered to express Wnt16, significantly increased trabecular bone volume/tissue volume and trabecular number (74). These findings are in consistent with human GWAS studies performed by different groups demonstrating the association between Wnt16 and spine/heel (predominantly trabecular bone) BMD (54, 67, 75). While no significant changes were observed in trabecular bone in the life-long Wnt16 inactivation mouse models used in previous studies, a recent study demonstrated that high-dose-tamoxifen-inducible Wnt16 inactivation modestly increased trabecular bone volume fraction in young adult mice (73). Moreover, Wnt16 knockdown has been shown to significantly decrease trabecular bone number in zebrafish (63). It is speculated that there may be a compensatory mechanism in mouse trabecular bone to maintain overall bone strength following reduction in cortical bone thickness (73). To date, possible reasons for the drastic differences in cortical bone phenotypes in Wnt16 knockout mouse models versus predominantly trabecular bone phenotypes in Wnt16 overexpression mouse models include (1): interactions of complex signaling pathways among several Wnts and their intracellular and extracellular modulators, (2) differences in bone micro-environments, (3) influences of mechanical signals perceived by the tissue, (4) cross-talk of Wnt signaling with other signaling pathways, and (5) large differences in physiological Wnt16 expression levels (high expression in cortical bone but very low expression in trabecular bone) (58, 59). Further studies are necessary to decipher the complex regulation of Wnt16 on trabecular bone, especially in physiological conditions.

Wnt16, which is highly expressed in cortical bones, is mainly produced by osteoblast-lineage cells, especially early/differentiating osteoblasts. The contribution of osteocytes to Wnt16 production is relatively small and bone marrow cells barely express Wnt16 (56, 60, 76). Wnt16 expression decreases during aging, which may be a possible cause of age-related bone loss (77). In cartilage of both humans and mice, Wnt16 is not expressed under normal conditions but is found to be rapidly upregulated in degenerated articular cartilage areas following injury or in OA (62, 78, 79). As reported in a recent study, Wnt16 expression is robustly increased under the stimulation of oncostatin M (a cytokine member of the IL-6 family) and then acts as a negative feedback regulator of oncostatin M-induced osteoclast formation (76). These findings indicate that the expression of Wnt16 is cell specific, and Wnt16 expression levels are important for many physiological and pathological conditions in which Wnt16 plays a role as a protective factor.

**Figure 2:**
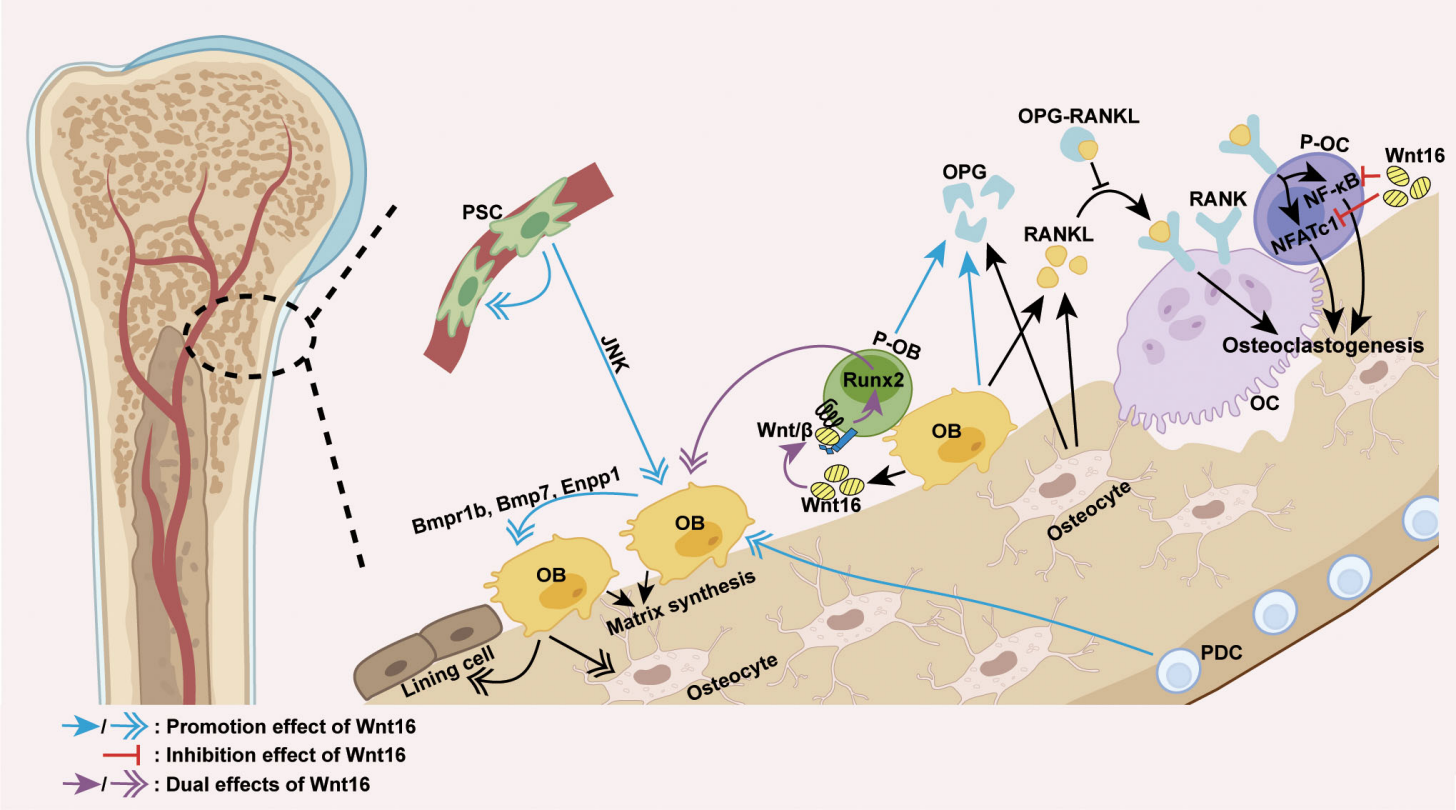
Wnt16 regulation of osteoblast differentiation and osteoclastogenesis. The predominant source of Wnt16 is the differentiating osteoblast. Wnt16 promotes osteoblast differentiation of PDCs, induces PSC proliferation, and increases osteoblast differentiation of PSCs in a JNK pathway-dependent fashion (74, 80, 81). By activating/repressing Wnt/β-catenin signaling, Wnt16 regulates Runx2 expression to promote/inhibit osteoblast differentiation of pre-osteoblasts (60, 82). In osteoblasts, Wnt16 upregulates genes involved in osteoblast differentiation and proliferation (*Bmpr1b*, *Bmp7* and *Enpp1*) (83). By inhibiting RANKL-induced NF-κB and NFATc1 signaling, Wnt16 directly inhibits osteoclastogenesis in osteoclast precursors. Moreover, Wnt16 indirectly inhibits osteoclastogenesis by upregulating the expression of OPG in pre-osteoblasts and osteoblasts, which binds to RANKL and thus inhibits the RANK-RANKL interaction (56, 84). PSC, perivascular stem cell; PDC, periosteal derived cell; OB, osteoblast; P-OB, pre-osteoblast; OC, osteoclast; P-OC, osteoclast precursor; H-chondrocyte, hypertrophic chondrocyte; Wnt/β, Wnt/β-catenin signaling.

Mechanically, the effect of Wnt16 on bone homeostasis appears to be primarily dependent on its regulation of osteoblast differentiation and osteoclastogenesis (**Figure 2**). The positive effect of Wnt16 on bone mass can be explained by its role in promoting osteogenic differentiation and inhibiting osteoclastogenesis. Recent studies have shown that recombinant Wnt16 treatment in osteoblasts upregulates many genes involved in osteoblast differentiation and proliferation, including *Bmpr1b*, *Bmp7*, and *Enpp1* (83). Wnt16 is capable of inducing perivascular stem cell (PSC) proliferation and enhancing PSC differentiation towards osteoblasts in a c-Jun N-terminal kinase (JNK) pathway-dependent fashion (80, 81). Similarly, Wnt16 promotes osteoblast differentiation from periosteal derived cells (PDCs) (74) as well as the differentiation of osteoblast progenitors into bone matrix-synthesizing osteoblasts by inhibiting canonical Wnt activity and upregulating Runx2 expression (82). However, it is worth noting that Wnt16 can also promote canonical Wnt activity to inhibit Runx2 expression and repress the differentiation of pre-osteoblasts (60). With regards to effects on osteoclastogenesis, Wnt16 has been shown to directly inhibit osteoclastogenesis by inhibiting RANKL-induced NF-κB and NFATc1 signaling in OPG-negative bone marrow-derived macrophages (osteoclast precursors) *in vitro*, and indirectly inhibits osteoclastogenesis by inducing the expression of OPG in a mouse pre-osteoblasts cell line (MC3T3-E1 cells) (56, 84).

Except for its role in promoting the expression of OPG in pre-osteoblasts, the effect of Wnt16 on other cell functions is insufficient to explain its regulatory effect on bone mass. Wnt16 has been shown to exert no effect on the functions of osteoclasts (56, 84). In addition, reduced periosteal bone formation caused by inducible Wnt16 inactivation during adulthood is known to result from a reduction in the number of cells rather than a lower activity per cell (73). Notably, the complexity and uncertainty of Wnt16 regulation on cell function has been highlighted by several other studies, which show that Wnt16 suppresses osteoblasts maturation and mineralization (56, 83) and life-long Wnt16 inactivation can lead to a robust reduction in mineral apposition rate, suggesting reduced cell activity (72). These findings indicate that Wnt16 is a powerful regulator of cell differentiation but not of cell function in bone homeostasis. The regulation of bone cell function may be facilitated by the coordinated regulation of multiple signaling pathways and/or downstream effectors triggered by Wnt16 in presence of specific receptors, co-receptors, antagonists and agonists.

**Figure 3:**
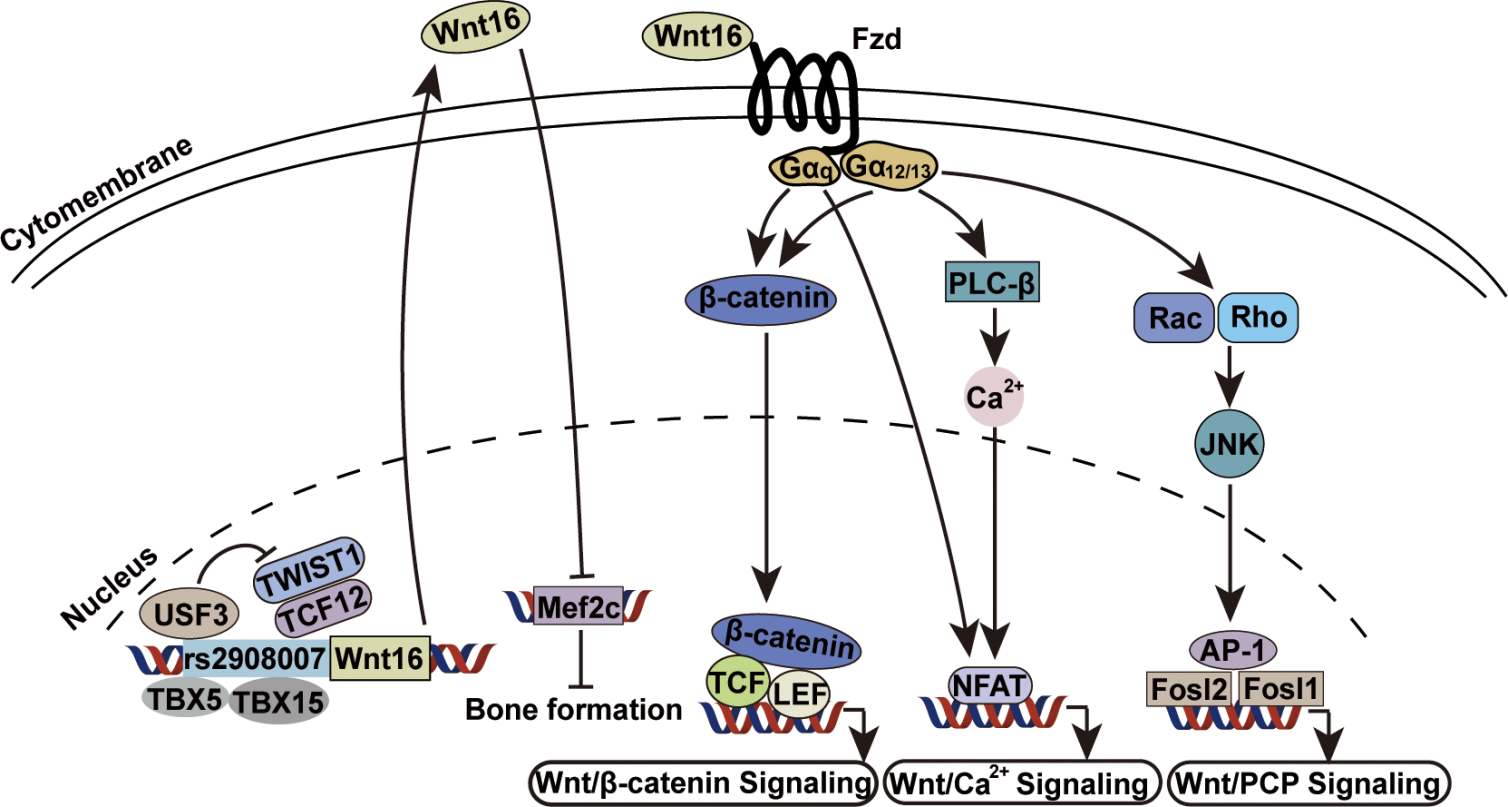
Wnt16 signaling transduction mechanisms. USF3, TBX5 and TBX15 bind to rs2908007[G] allele to increase Wnt16 expression. The repression of Wnt16 by anti-osteogenic TWIST1 and TCF12 is antagonized by USF3 (88). Wnt16 protein transcriptionally represses Mef2c which is a known negative regulator of bone formation (83). Gα12, Gα13 and Gαq are required as intracellular partners by Wnt16 for Wnt/β-catenin signaling, Wnt/Ca2+ signaling and Wnt/PCP signaling (87). Gα12, Galpha 12; Gα13, Galpha 13; Gαq, Galpha q.

Wnt16 can activate both canonical and non-canonical Wnt signaling in osteoblasts and chondrocytes (56, 85), but can only signal through the non-canonical pathway in osteoclasts (56, 79). Identified Wnt16 receptors include AP2b1, Ror2 and CD146 of the planar cell population (PCP) and JNK pathway (62), and LRP6 of the canonical pathway (56). However, the specific receptor of Wnt16 in the skeletal system remains unclear, although a recent study showed that a non-canonical Fzd1/2/7 receptor may be Wnt16-specific during early anterior-posterior patterning and morphogenesis for knockout of the Fzd1/2/7 receptor produced the same phenotype as Wnt16 knockout (86). Further cellular and molecular analyses have shed light on the specific intracellular partners and transcription factors involved in Wnt16 signaling. Galpha subunits (including Gα12, Gα13, and Gαq) are intracellular partners of Wnt16 required for both canonical and non-canonical Wnt signaling activity in osteoblasts (87). In addition, rs2908007 (an osteoporosis GWAS variant located in the Wnt16 promoter) confers G-allele-specific transcriptional modulation *via* TBX5/TBX15, USF3, and TWIST1/TCF12. TBX5 and TBX15 bind to the rs2908007 allele to increase Wnt16 expression, and USF3 transactivates Wnt16 promoter activity and antagonizes the repression of Wnt16 by TWIST1 and TCF12, thereby contributing to the enhancement of osteoblast differentiation and the suppression of osteoclastogenesis in cultured human osteoblast-like U-2OS cells (88). Moreover, Mef2c, a negative regulator of bone formation, has been identified as the most significantly enriched transcription factor associated with genes repressed by Wnt16, while Fosl2 and Fosl1 (JNK pathway activated transcription factors) are two of the most significantly enriched transcription factors associated with genes activated by Wnt16 (**Figure 3**) (83). The discoveries of these specific factors undoubtedly add details to the big picture of the Wnt16 signaling network.

The Wnt16 signaling network is complex, as Wnt16 signals crosstalk with other signaling pathways. Analysis of the Wnt16-regulated transcriptome has revealed that genes regulated by Wnt16 in osteoblasts include members of the Wnt pathway/family, mTOR signaling pathway and other pathways. A large number of Wnt16 targets overlap with the canonical Wnt3a targets and non-canonical Wnt5a targets (83). This crosstalk was confirmed in another study using protein-protein interaction network analysis (63). Functional experiments support the interactions of Wnt16 with Wnt5a and Wnt3a. The inhibitory effects of Wnt16 on RANKL-induced osteoclastogenesis can be abrogated by Wnt5a; however, Wnt16 is unable to inhibit the Wnt5a-induced expression of RANK in osteoclast precursors, indicating that Wnt5a and Wnt16 strictly coregulate osteoclastogenesis in a manner dependent on various conditions (84). Peptide-Wnt16 mRNA nanocomplexes can antagonize canonical β-catenin/Wnt3a signaling in human cartilage explants, demonstrating the interaction between Wnt16 and Wnt3a (89). In addition, it has been shown that Wnt16 forms a network with other genes by forming connections with the two genes *sulf1* and *vegfaa* (63). Together, these findings indicate that the different upstream signals may product the same effect, and the downstream effects of Wnt16 signaling can differ in different bone microenvironments. As we deepen our understanding of the Wnt16 signaling network, the initial etiology and corresponding therapeutic targets of complex bone diseases will become clear.

## Wnt16 in osteoarthritis pathogenesis

While previous work indicated that there was no association between Wnt16 gene polymorphisms and osteoarthritis (OA) (90), recently, two SNPs of the Wnt16 gene (rs2707466 and rs2908004) known to be associated with BMD have been implicated in the hip and knee OA phenotypes in Caucasian patients in a sex-dependent manner (91). This report was the first to suggest the involvement of the Wnt pathway in determining the OA phenotypes. Furthermore, a more recent study revealed that two SNPs of Wnt16 (rs2908004 and rs1799986) are associated with decreased risk of OA, implying that Wnt16 plays a protective role in the pathology of OA (92). Supporting the functional role of Wnt16 in limiting cartilage destruction in OA are the findings that mice with global Wnt16 deletion (Wnt16^-/-^ mice) or chondrocytes-specific (Col2a-cre) Wnt16 deletion develop more severe experimental OA after OA-induced surgery, which is charactered by deteriorated articular cartilage integrity, an increased subchondral bone remodeling rate, more severe synovitis, prominent osteophyte formation, and upregulated MMP13 and Col10a1 (62, 79, 93). Altogether, these new findings support the vital role of Wnt16 in OA, although larger-size studies and studies including other patient populations are still needed to validate these findings on a broader scale.

**Figure 4:**
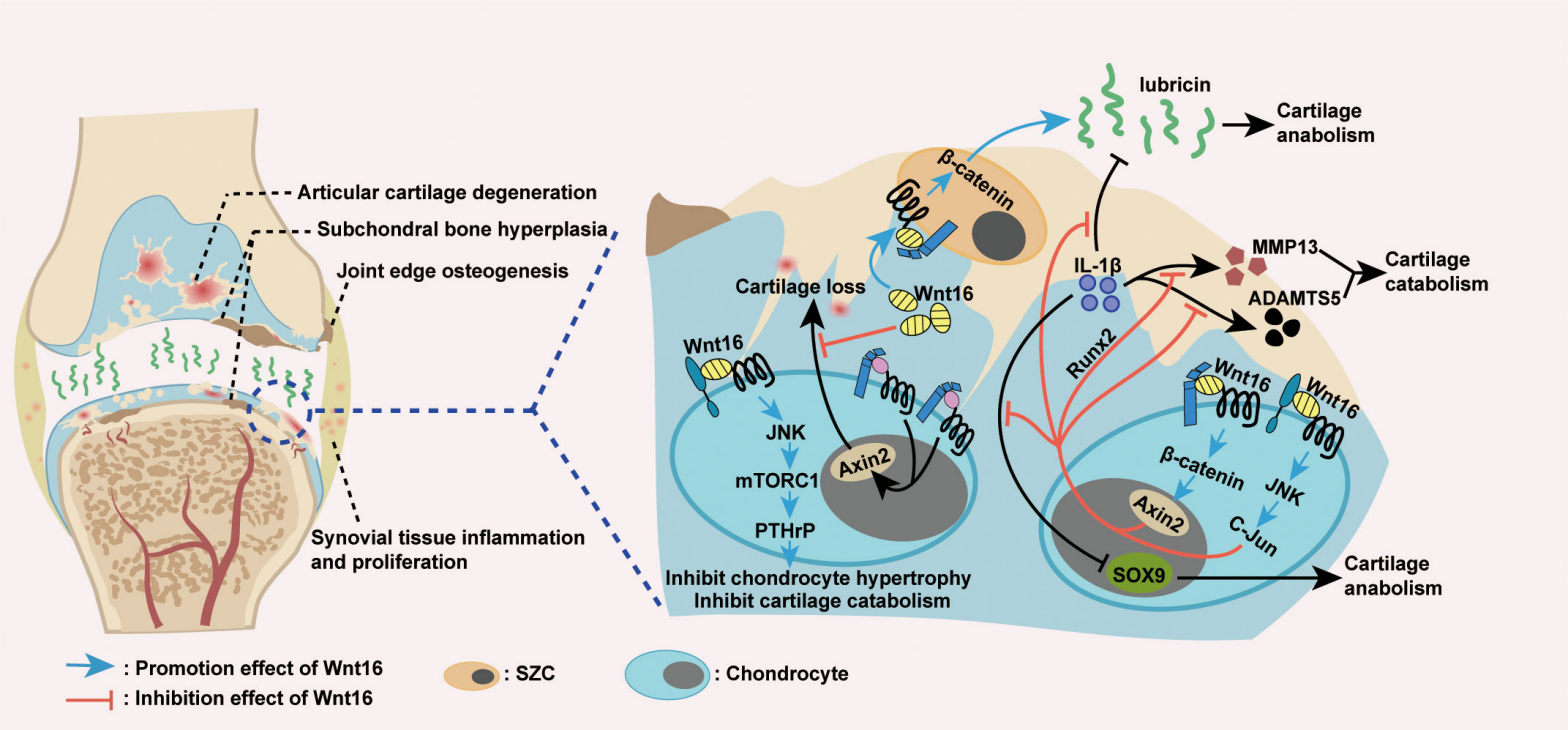
Wnt16 regulation of OA. Excessive activation of Wnt/β-catenin signaling causes cartilage loss, while Wnt16 protects cartilage by antagonizing over-activated Wnt/β-catenin signaling (79, 94). Wnt16 attenuated IL-1β induced suppression of cartilage anabolic factors SOX9 and lubricin and IL-1β induced activation of cartilage catabolic factors MMP13 and ADAMTS5, probably through Wnt16/β-catenin and Wnt16/JNK signaling pathways. Also, Wnt16’s suppression of MMP13 under inflammatory conditions is the result of Wnt16 inhibiting IL-1β induced Runx2 expression (85). Wnt16 supports the phenotype marker profile of superficial zone cells (SZCs) and may upregulate lubricin expression partially through Wnt16/β-catenin signaling (79, 85, 89). Through Wnt16/JNK-mTORC1-PTHrP cascade, Wnt16 inhibits chondrocyte hypertrophy and thus inhibits cartilage catabolism (62). IL-1β: Interleukin-1β; MMP13: Matrix Metallopeptidase 13; ADAMTS5: a disintegrin and metalloproteinase with thrombospondin motifs 5.

From a mechanistic point of view, the protective effect of Wnt16 on joints in OA is a result of its inhibition on cartilage catabolism (**Figure 4**). The first evidence for this effect was the finding that, in mouse temporomandibular joint (TMJ) cartilage, Wnt16 not only attenuates IL-1β-induced suppression of cartilage anabolic factors (including SOX9 and Lubricin), but also suppresses the expression of IL-1β-induced cartilage catabolic factors (including MMP13 and ADAMTS5). Mechanistically, Wnt16 has been shown to signal through both the canonical Wnt/β-catenin and non-canonical Wnt/JNK-cJUN pathways and exert protective effects *via* Runx2-mediated suppression of MMP-13 in TMJ fibrochondrocytes under inflammatory conditions (85). Furthermore, during OA pathogenesis, Wnt16 suppresses chondrocyte hypertrophy, thereby contributing to a reduction in Col2a1 (the major component of the cartilage matrix), *via* PCP/JNK-mTORC1-PTHrP cascade (62). Moreover, Wnt16 may compete with other, more effective Wnts (such as Wnt3a and Wnt8) to limit the over-activation of the canonical Wnt pathway, which is known cause of severe OA in Wnt16-deficient mice, thereby protecting cartilage in OA (79, 94). Collectively, Wnt16 inhibits cartilage catabolism in OA by inhibiting IL-1β-induced inflammation, suppressing chondrocyte hypertrophy, and antagonizing over-activated canonical Wnt signaling.

Wnt16 also protects joints by maintaining or inducing cartilage anabolism during OA pathogenesis (**Figure 4**). In addition to the excessive activation of Wnt/β-catenin signaling, severe OA in Wnt16^-/-^ mice was associated with the reduced expression of lubricin (an essential anabolic factor) and increased apoptosis of articular cartilage superficial zone cells (SZCs), which contribute to lubricin production and articular cartilage self-renewal (79). *In vivo*, as mentioned above, Wnt16 can upregulate lubricin expression during inflammation induced by IL-1β (85). *In vitro*, Wnt16 not only upregulates lubricin expression in primary bovine articular chondrocytes and human cartilage explants, but also supports the phenotype marker profile and upregulates lubricin production in SZCs (79, 89). Mechanistic studies have shown that high doses of Wnt16 strongly upregulate lubricin by weakly activating canonical Wnt signaling (79). Overall, Wnt16 promotes cartilage anabolism in OA by upregulating the expression of lubricin and supporting the phenotype marker profile of SZCs.

## Effects of local Wnt16 treatment *in vivo*

As a key regulator of skeletal homeostasis and OA progression, Wnt16 has great potential to serve as a locally delivered molecule for treating skeletal disorders. Indeed, the inherent difficulties in stabilizing and delivering the hydrophobic Wnt protein as a bioactive reagent *in vivo* have been overcome——liposomes are effective delivery vehicles of preserving both *in vitro* and *in vivo* Wnt proteins stability and activity (95, 96). Current research on the effect of local Wnt16 treatment focus on three major aspects: Wnt16 application in healthy bones and joints, in OA models, and in bone defect models.

In the Wnt16-treated healthy tibia, local BMD is significantly higher as compared with the contralateral tibia treated with empty liposomes, consistent with the genetic finding that Wnt16 is a positive regulator of BMD (56) and indicating that local application of Wnt16 can increase bone mass. Although there are doubts about whether high BMD is a risk factor for OA (97–102), a recent study demonstrated that high bone mass resulting from Wnt16 overexpression was not associated with more severe OA, suggesting that bone mass is not the main contributor to OA severity and treatment strategies targeting the regulation of Wnt16 are unlikely to increase the risk of developing OA (103). However, another study unexpectedly found that Wnt16 application may have a negative effect on cartilage in healthy joints: in joints without OA symptoms, intra-articular injection of adenovirus-Wnt16 resulted in an increase in canonical Wnt signaling and led to superficial erosive lesions and several other lesions in cartilage (104). Therefore, we suggest that local Wnt16 application, especially in health sites, should not be applied until there is sufficient supporting evidence.

Under inflammatory conditions, local application of Wnt16 in the articular cavity was reported to ease the severity of OA. It has been reported that intra-articular injection of adenovirus-Wnt16 into mouse knee joint cavities significantly improved all OA parameters and attenuated cartilage degradation, while Wnt16 knockdown mice exhibited more severe OA after anterior cruciate ligament transection surgery (62).

In a mouse model of bone defect, Wnt16 delivered in liposomes prevented bone loss, demonstrating that Wnt16 has the capacity to improve bone status *in vivo* (56). In another study, after transplantation of PDCs infected with Wnt16-containing virus supernatant into a mouse calvarial defect model, new bone formation was observed in the defect area, suggesting that local Wnt16 application may induce bone formation (74).

Taken together, evidences to date indicates that, Wnt16 treatment in the joint cavity can ease OA, and local application of Wnt16 in bone defect sites is beneficial for preventing bone loss and inducing bone formation. However, it is worth noting that the application of Wnt16 in healthy joints may have risks. Also, current animal models for studying the effect of local Wnt16 treatment *in vivo* are still limited to mouse and rat models. Additional animal and clinical experiments together with long-term efficacy trials of local Wnt16 treatment are required to progress the development of this putative treatment strategy.

## Conclusions and perspectives

In this review, we provided new insights regarding the role of Wnt16 as a regulator of both cortical and trabecular bone homeostasis and discussed the role of Wnt16 as a protective regulator in OA. In addition to clarifying these roles of Wnt16, we also emphasized the precise mechanisms regulating Wnt16 signaling, including its role in the regulation of skeletal development, postnatal management of bone hemostasis independent of developmental influences, mechanisms on osteoblastogenesis and osteoclastogenesis, and multidimensional protective mechanisms in OA. Notably, in animal models, the local application of Wnt16 in inflamed joints as well as in bone defect sites exhibit exciting therapeutic effects. With an increasing number of transcription factors and crosstalk signals still to be discovered, a broader understanding of Wnt16 signaling will bring more insights and greater opportunities for the targeted therapy of bone diseases.

The spatiotemporal expression of Wnt16 has been described by several studies: during embryonic development, Wnt16 is selectively expressed in mouse perichondrium, periosteum and joints, and in zebrafish dermomyotome and developing notochord (105, 106), while in postnatal environment, Wnt16 is highly expressed in mouse cortical bone and zebrafish notochord sheath (56, 105). Researches have suggested that changes in hormone level caused by aging, diseases and hormone therapy can affect the expression of Wnt16. Ovariectomy or estrogen deficiency has shown to decrease Wnt16 expression, whereas estradiol and Tamoxifen treatment increase Wnt16 expression in mouse cortical bone (59, 77). In addition, glucocorticoid treatment suppresses Wnt16 expression, probably *via* direct DNA-binding mechanisms (107–109). However, how hormones affect the spatiotemporal expression of Wnt16 and how Wnt16 coregulate bone homeostasis with hormones have not been clearly clarified. Although Wnt16 restored glucocorticoid-induced suppression of bone formation and protected against glucocorticoid-induced bone loss in two studies (107, 108), one study showed that upregulation of Wnt16 expression was insufficient to prevent glucocorticoid-induced bone loss in mice (109). Moreover, it is shown that the bone-sparing effects of estrogen and Wnt16 are independent of each other (59). Therefore, much more knowledge is needed regarding the spatiotemporal expression of Wnt16 and its association with hormones in physiological and pathological conditions to truly open novel avenues for the therapy of a variety of skeletal diseases.

## Author contributions

XL designed and formulated the review theme. XY searched and reviewed literature drafted manuscript and revision; XL provided critical comments, discussed and revised the manuscript. All authors contributed to the article and approved the submitted version.
